# Development of a comprehensive clinical assessment protocol for low back and hip pain in powerlifters: a feasibility study

**DOI:** 10.1186/s40814-024-01579-0

**Published:** 2024-12-12

**Authors:** Patrik Olofsson, Ulrika Aasa, Lars Berglund

**Affiliations:** 1 Laponia Health Care Center, Norrbottens County Council, Gällivare, Sweden; 2 Care Sciences and Society, Physiotherapy, Karolinska Institutet, and Karolinska University Hospital, Medical Unit Allied Health Professionals, Stockholm, Sweden; 3 Department of Community Medicine and Rehabilitation, Physiotherapy, Umeå University, Umeå, Sweden

**Keywords:** Physical therapy, Clinical assessment protocol, Powerlifting, Low back pain

## Abstract

**Background:**

Low back and hip pain problems are frequent in powerlifting. There is a lack of information on the specific pain phenotypes and specific impairments in body function associated with these pain problems, as well as how to reach a clinical diagnosis relevant for powerlifters’ pain problems. Therefore, the aim was to develop a comprehensive clinical assessment protocol for pain and function in powerlifters with low back and/or hip pain and evaluate its feasibility for use in further epidemiological or clinical studies of powerlifters.

**Methods:**

The study was conducted in two phases. In phase one, the protocol was developed according to literature regarding musculoskeletal assessment and sports injuries, and in phase two, feasibility was evaluated. Eight powerlifters with low back/hip pain were included in phase two. Four of them were examined independently by two physical therapists, and the other four were examined by one of the physical therapists. The time spent on the examination, whether the physical therapists could reach a clinical diagnosis without adding items to the protocol, and whether the two physical therapists were consistent in terms of the clinical diagnosis, were evaluated.

**Results:**

The protocol was developed including subjective examination, physical examination, and a clinical diagnosis based mainly on signs and symptoms of associated neurophysiological pain mechanisms and the specific impairments in body functions associated with the powerlifter’s pain problem. The protocol met the feasibility criteria. The examination lasted approximately 1 h, no items needed to be added, and both physical therapists were able to make a consistent clinical diagnosis. Visual observation and alteration of movement strategy of the squat/deadlift were thought to be crucial for assessing the powerlifters’ pain problem.

**Conclusions:**

This is the first comprehensive clinical assessment protocol developed to describe powerlifters’ pain problems/injuries with a clinical diagnosis based on the dominating neurophysiological pain mechanism and impairments in body functions. However, before use in larger studies, it is recommended that the protocol be further evaluated by a larger number of physical therapists and powerlifters to evaluate its reliability and whether the content of the protocol should be further expanded.

**Supplementary Information:**

The online version contains supplementary material available at 10.1186/s40814-024-01579-0.

## Key messages regarding feasibility

- What uncertainties existed regarding the feasibility?

No previous study has developed a comprehensive clinical assessment protocol specifically for low back and hip pain in powerlifters, nor evaluated the feasibility of its components for clinical use in establishing a diagnosis that could guide treatment.

- What are the key feasibility findings?

The protocol proved feasible for clinical assessment, as it could be completed within an acceptable time frame, and its components enabled the therapists to consistently reach an informative clinical diagnosis regarding the powerlifters’ pain presentation and associated impairments in body function.

- What are the implications of the feasibility findings for the design of the main study?

For future studies seeking to examine, characterize, or treat low back and/or hip pain in powerlifters, including their specific impairments in body function, the protocol was feasible and is therefore applicable for further research and clinical evaluation.

## Introduction

Pain problems and/or injuries to the low back and hip regions which hinder continuous training are common in powerlifting [1]. Since all previous research on the prevalence and incidence of pain problems/injuries has been performed through questionnaire studies [2], they have never been further described and classified in terms of their dominating pain mechanism[3] and impairments in body functioning (as defined by the International Classification of Functioning, Disability and Health by the World Health Organization) [4]. The Van Mechelen sequence of prevention for musculoskeletal injuries states that injuries need to be thoroughly characterized before preventative strategies may be suggested [5]. Thus, to characterize pain problems and/or injuries in a way that is also relevant for their treatment, a comprehensive standardized assessment protocol is needed.

Presently, there is no consensus on how to describe and define the characteristics of sports injuries and/or pain problems [6]. To date, sports injuries are defined in several ways, for example, by their onset (acute onset/traumatic or gradual onset/overuse), duration/phase of symptoms (acute, sub-acute, or chronic), or by the impact of the injury on their ability to continue with their sport [6]. None of the currently used definitions provide the necessary information to guide treatment or prevention, which is why researchers in sports medicine have requested methodological studies regarding the clinical classification of gradual onset overuse/chronic pain problems and/or injuries [7].

In clinical examination, therapists rely on a clinical reasoning process to assess the signs and symptoms of a patient’s pain problem based on history taking and investigation of physical functioning [8]. In sports medicine, additional examinations of posture and movements during sport-specific activities are often included. The examination process can be divided into four parts with different aims. First, diagnostic triage is undertaken with the aim of identifying signs of serious pathology (red flag disorders), radicular syndrome, or pain arising from other causes (for example diseases) [8]. Second, if serious pathology is ruled out, it is determined whether the pain problem is in an acute, sub-acute, or chronic stage as well as which neurophysiological pain mechanism is dominant [3, 9]. By assessing signs and symptoms to determine the dominating pain mechanism it can be established whether the pain is driven by increased signaling from the central or peripheral nervous system or by activation of nociceptors as in inflammatory, mechanical, or ischemic pain [3, 8, 9]. Third, it is assessed whether the condition is adaptive, i.e. protecting tissues to stimulate healing, or maladaptive, i.e., a condition where either thoughts, feelings, posture, or movement behaviors are associated with the condition [10]. Fourth, an examination of the patient’s physical functioning based on the assessment of movement capacity, movement strategies, and symptoms during exposure to certain activities and movements is performed [11]. The purpose of the examination process is to provide the basis for a diagnosis that will also guide treatment, i.e., whether the patient will benefit from rest, gradual exposure to load, specific treatment of neurological symptoms, re-training of movement strategies, cognitive interventions, or a combination of strategies in order to treat the pain problem/injury.

A final diagnosis provided by a therapist can be formulated in many ways as several approaches exist for this purpose. Some researchers and clinicians argue that examination and diagnosis based on symptom and movement assessments are more direct to the subsequent treatment than a patho-anatomic diagnosis, where the pain problem is classified by determining the anatomical structure responsible for pain and dysfunction [12]. This is especially true for gradual onset and chronic pain problems. Furthermore, in a review by Karayannis et al. [11], it was concluded that movement-based approaches have higher reliability than a patho-anatomic approach, although the validity of each approach has been difficult to determine.

Since gradual onset overuse pain problems seem common in powerlifting [1] and athletes and experts in this area [1, 13] attribute these to current lifting technique, a movement-based diagnosis that considers individual lifting techniques, and the possible association to the pain problem, could be critical for understanding both causative mechanisms and mechanisms of treatment response. Furthermore, there is a lack of evidence for specific risk factors for injuries in powerlifters related to impairments in body function or training history[1, 2, 14], leaving no clear guidance for developing evidence-based assessment protocols. As a result, the development of an assessment protocol in this context must instead rely on general principles and expert opinion.

Powerlifting is a sport where athletes compete with the aim of lifting the most weight in the squat, bench press, and deadlift [15]. Training is generally very monotonous and usually consists of performing the exercises, and variations thereof, several times a week with moderate to high loads. In the squat, muscles around the ankle, knee and hip, and spine support and move the load [16]. Similar demands are presented in the deadlift [17], although it generally places a greater proportion of the load on the hip and spine. Both exercises place high demands on tissue strength and movement control, especially for the hip and spine, which are often recommended to be kept in a neutral position (i.e., midrange position) [13]. In the bench press, muscles of the upper body primarily support and move the load, and movement control of the shoulder, scapula, and spine is necessary to create a stable base and precise movement of the bar [18]. Although the physical demands of the exercises are somewhat generalizable, given the varied body compositions, mobility, and anthropometry of powerlifters, there is also a diverse range of successful lifting techniques used in the respective exercises.

Given that powerlifting training involves complex movements with a high load performed on a frequent basis, it stands to reason that a physical examination of pain problems in powerlifting should involve a thorough analysis of the lifting technique. Like physical examination of non-sport-related pain problems, the examination should strive to determine a clinical diagnosis and treatment strategy for the pain problem through examination of the associations between physical and psychological function and symptoms. To perform the examination, assessment, and diagnosis of pain problems and/or injuries in powerlifting, a standardized comprehensive clinical assessment protocol is necessary. The aim of the protocol should be to describe the powerlifters’ pain problem in a way that provides meaningful information regarding current symptoms and specific impairments in body function [7].

Therefore, the aim of this study was to develop a comprehensive clinical assessment protocol for pain and function in powerlifters with low back and/or hip pain and evaluate its feasibility for use in further studies aiming to describe prevalence of different pain problems/injury types, evaluate treatments or investigate the risk of injuries, in powerlifting. To evaluate feasibility, the following objectives were formulated: (1) to determine the time required to apply the clinical assessment protocol; (2) to assess whether the clinical assessment protocol is comprehensive enough to establish a clinical diagnosis, or if there are missing items; (3) to assess the reliability between two independent assessors of the assessments and classifications of pain problems; (4) to identify any unnecessary items from the clinical assessment protocol that do not contribute to reaching a clinical diagnosis; (5) to investigate whether altering movement strategies during provocative movements (e.g., squats or deadlifts) can reduce self-reported symptoms.

## Method

### Design

The present study was a design and feasibility study of a comprehensive clinical assessment protocol for pain and function in powerlifters with low back and/or hip pain. The study was performed in two phases. Phase one included an assessment of the literature regarding musculoskeletal assessment and sports injuries and the development of the protocol. In phase two, feasibility was evaluated by two physical therapists (PTs) examining powerlifters with low back and/or hip pain.

### Ethical considerations

The study was approved by the regional ethical review board in Umeå, nr. 2014–285-31 M. All powerlifters provided written consent after being informed about the contents of the study. Powerlifters were informed that there were no known risks of participating other than those normally present while attending a physical therapy session and about their right to cancel participation at any time.

### Phase one—development of the comprehensive clinical assessment protocol

Initially, three physical therapists (PO, UA, LB) reviewed the scientific literature on powerlifting injuries and the classification of sports injuries [2, 6, 7], and the curricula of physical therapy programs in Sweden, and postgraduate courses in examination and classification of musculoskeletal disorders [8, 19–22], including the International Federation of Manual and Musculoskeletal Physical Therapists Incorporated (IFOMPT) postgraduate specialization educational standards [23]. Based on the literature, they identified best practices and drafted the first protocol. The protocol included (1) a subjective examination (history taking) focusing on the powerlifters’ pain experience and activity limitations and (2) a physical examination with defined tests. The testing involved observation of posture, peripheral nerve tests, examination of specific pain provocative activities reported by the powerlifter including the squat and deadlift exercises, pain provocation and range of motion tests as well as test of muscle function and motor control. The purpose of the physical examination was to collect data on body functions with possible association with the powerlifter’s pain problem to guide diagnosis. And (3) a clinical diagnosis based on signs and symptoms related to neurophysiological pain mechanisms and the specific impairments in body functions associated with the powerlifter’s pain problem.

After completion of the protocol, two PTs specializing in orthopedic manual therapy and sports injuries were asked to review the protocol regarding the relevance of documenting the clinical reasoning process to determine a pain and movement-based clinical diagnosis. The results of the input from the expert clinicians are shown in Table 1.

**Table 1:** Feedback on the first draft of the protocol from two expert physical therapists

“The examination should be general and broad, not too specific (with various tests) in the first step, as it is challenging to know which tests/examinations are relevant to perform on powerlifters”	PT #1
“Segmental pain provocation test for the low back should complement the protocol. For example springing test?”	PT #2
“Distinguishing between stiffness versus structural tightness in a muscle can be challenging. There are no clear guidelines on this.”	PT #1 and 2
“Be cautious with specific movement control tests initially, as it is difficult to determine what is relevant for powerlifters.”	PT #2

### Phase two—evaluation of feasibility

In phase two, the feasibility of the final protocol (Supplemental File 1) was assessed.

#### Participants

For the evaluation of feasibility, two PTs with a specialization in orthopedic manual therapy (i.e., a post-graduation specialization in neuro-musculoskeletal disorders for physical therapists) and > 8 years of experience of working in primary care were included. The powerlifters were recruited through a convenience sample from local powerlifting clubs in northern Sweden. Powerlifters who currently experienced pain/discomfort in their low back or hip for at least 6 weeks while performing of the squat and/or deadlift and who were currently experiencing associated limitations in their training or competition were eligible for inclusion. Powerlifters with any signs of red flag pathologies [8] or acute pain (< 6 weeks) were excluded.

In total, ten powerlifters were recruited, whereas two dropped out before the physical examination (due to logistical/time concerns), leaving eight powerlifters to be included in the study. For detailed background information of the included powerlifters, see Table 2.

**Table 2:** Background characteristics of powerlifters. Median, minimum, and maximum values presented

	**Male (** ***n*** ** = 5)**	**Female (** ***n*** ** = 3)**
Age (years)	29 (24, 50)	26 (26, 29)
Weight (kg)	93 (83, 115)	66 (59, 74)
Height (cm)	174 (168, 188)	165 (165, 168)
Powerlifting experience, training (years)	10 (7, 30)	3 (2, 4)
Number of training sessions per week—squat (*n*)	3 (1, 4)	2 (1, 4)
Number of training sessions per week—bench press (n)	3 (1, 4)	3 (1, 5)
Number of training sessions per week—deadlift (*n*)	2 (1, 4)	2 (1, 2)
Training hours per week (h)	10 (6, 12)	7 (3, 12)
Personal best—squat (kg)	185 (170, 262.5)	125 (85, 127.5)
Personal best—bench press (kg)	165 (140, 203.5)	70 (55, 85)
Personal best—deadlift (kg)	230 (200, 300)	156 (110, 160)

#### Sample size

To evaluate the feasibility goals of the protocol itself, in a relatively homogenous population, it was deemed that eight powerlifters were sufficient. Also, previous studies have been published using similar sample sizes to answer similar research questions on feasibility. For example, when developing a training and injury log [24] or proving the success of study data collection protocol in a biomechanics study [25].

#### Procedure

The protocol was evaluated by the PTs while examining the powerlifters with low back and/or hip pain according to the protocol. Four of the powerlifters were assessed by both PTs with a 15-min interval between the examinations. The other four were assessed by one of the PTs. All examinations and assessments were performed independently, i.e., there was no collaboration between the PTs during/between examinations. The PTs took notes on the protocol as they performed the examination. The equipment used for the examination was a stopwatch, examination table, and a barbell (20 kg) with two 10-kg weight plates.

#### Feasibility criteria

Based on the research questions regarding the feasibility of the protocol, the following criteria for feasibility were set: the examination needed to be completed within 60 min, the PTs should be able to arrive at a clinical diagnosis without adding any additional items to the protocol, and it should be possible to modify the powerlifters’ low back/hip pain through alteration of movement strategy in the squat/deadlift in a majority of cases. Furthermore, when the two PTs assessed the same powerlifter, they should arrive at the same clinical diagnosis regarding the dominating pain mechanism and primary impairments in body function associated with the powerlifters’ pain problem, across all the assessed powerlifters. To assess these criteria, the examiners answered a “feasibility questionnaire” regarding all the respective feasibility criteria at the end of the assessment of each powerlifter.

#### Data analysis

Data regarding quantitative measures, i.e., the time of each assessment and whether symptoms could be altered during the assessment, were presented as median and minimum and maximum values and ratios as percentages. The data regarding the assessments performed using the protocol, both regarding different items and the final clinical diagnosis, were presented in a qualitative and descriptive manner and are displayed with citations from the PTs. Additionally, comments on suggested revisions to the protocol were also presented with citations.

## Results

### Phase one—development of the comprehensive clinical assessment protocol

The final version of the comprehensive clinical assessment protocol is presented in Supplemental file 1 and described henceforth.

#### Contents of the comprehensive clinical assessment protocol

Since the purpose of the protocol was to serve as the basis for a formalization of the clinical reasoning process, the therapist applying the protocol should be able to arrive at a final clinical diagnosis based on the signs and symptoms of associated neurophysiological pain mechanisms and the specific impairments in body functions associated with the pain problem experienced by the powerlifters. Therefore, the examination included a detailed subjective examination and a physical examination that included a wide range of tests of physical function [8]. There are many tests that can/should be included in the physical examination when assessing musculoskeletal disorders. In clinical contexts, findings in the subjective examination guide the choice of tests, but since there are no previous studies on the examination of powerlifters and their specific pain problems/injuries, it was not possible to determine in advance which specific tests should be included; therefore, a range of components that are typically included in physical examination of musculoskeletal pain/injuries were included in the protocol.

#### Subjective examination

The subjective examination (history taking) aimed to identify and map possible causes/sources of the patient’s symptoms; the patient’s experiences related to the symptoms, training history, and current training regime; and the presence and impact of potential psychosocial factors. Therefore, questions regarding the location of pain, debut, duration of pain, and pain behavior were included. Powerlifters were also asked to rate their current pain intensity as well as their pain intensity on average over the last seven days on a visual analog scale (VAS) 0–100 mm [26]. The VAS for pain intensity is considered a valid and reliable measure of pain intensity and is recommended for use in quantifying pain intensity in patients with low back pain [27]. Additionally, powerlifters were also asked to describe three activities that they experienced limited in performing due to their pain problem and also grade these activities on a scale of 0–10 (i.e. using the patient-specific functional scale (PSFS)[28] where “0” equals not being able to perform the activity due to the pain problem, and “10” equals no limitation in activity due to the pain problem. The PSFS was included to further describe the consequences of the powerlifter’s pain problem from a movement perspective and subsequently use it as an evaluation tool from baseline to follow-up visits.

Open-ended questions were also included to allow powerlifters to describe their pain problems in their own words and express their thoughts regarding the causes and consequences of their symptoms. The open-ended questions were posed in a way that required the patient to articulate their own response. Additionally, encouraging techniques, such as nodding in agreement or asking follow-up questions such as “What do you mean, can you elaborate?” were employed [29].

#### Physical examination

Experienced clinicians and researchers suggest that a physical examination of pain problems and injuries should involve elements that allow a clinical diagnosis that describes the patients’ activity limitations, dominating pain mechanisms, and impairments in body function related to their pain/injury [8, 9, 12]. Therefore, this was also the rationale when selecting the appropriate elements to make up the protocol, guiding the clinical reasoning process and leading to a pain mechanism and movement-based clinical diagnosis of the powerlifters’ pain problem.

The initial step involved observing and describing the powerlifter’s resting posture in a structured manner thereby guiding the PT in understanding of possible strategies to provoke or alleviate pain in upcoming examinations (see Supplemental file 1). Next, the peripheral nervous system was examined to confirm or rule out peripheral neuropathic pain.[3, 9] The tests included sensory testing to light touch, isometric muscle tests, reflexes, and mechanosensitivity assessments.[8, 30] For identifying lumbo-sacral radiculopathy, sensitivity and specificity have been presented as follows for sensory testing, sensitivity 0.08–0.61, specificity 0.58–1.00; isometric muscle tests, sensitivity from 0.13 to 0.61, and specificity 0.47–0.97; reflexes, sensitivity 0.14–0.67 and specificity 0.6–0.93 [30]. The straight leg raise (SLR) test, which has previously demonstrated acceptable inter-rater reliability, was used to assess mechanosensitivity.[31] The test has a sensitivity 0.29–1.00 and specificity 0.21–1.00 for identifying lumbo-sacral radiculopathy [30].

To assess the activities in which the powerlifters experienced limited due to their pain problem (as reported in the PSFS), including movement strategies and possible easing or provocative factors, the examination included an assessment of these activities. In this examination, the powerlifters were asked to perform the activities, whereas the examiner first observed the execution and if the powerlifter experienced any pain, the examiner manually and/or verbally attempted to alter the way that the powerlifter performed the activity. For example, if the powerlifter had pain in the low back when performing the back squat and the examiner also observed an exaggerated lumbar lordosis, the powerlifter was instructed to change their lumbar position and perform more repetitions and report any change in symptoms. This procedure is much alike the test/secondary test which has been systematized by, Sahrmann [19, 32] which gives the examiner an idea of possible associations between physical/biomechanical factors (such as movement control, flexibility, and strength) and the pain problem.

Thereafter, active and passive movement tests, including assessment of end-feel [8], were included for the lumbar spine and hips, while assessing any pain on movements as well as an assessment of the range of motion and flexibility, noting any differences between the left and right sides. This movement testing aimed to provide information regarding the location of pain, specific movements provoking the pain, and possible causes for movement restrictions [8] and their consequences for the powerlifter’s movement strategies during pain provocative activities. Also included, was a test of ante-/retroversion angle of the hip joints, Craig’s test [33], where the examiner assesses the neutral position of the hip joint, i.e. the position where the hip joint has an equal range of movement in internal and external rotation respectively. Craig’s test has previously demonstrated an excellent intra- and inter-rater reliability (intra-class correlation > 0.83) [34]. The test has also been validated against magnetic resonance imaging for establishing femoral version angle with a correlation coefficient of r = 0.61 [34]. For the low back, the springing test (also called the posterior/anterior test) was included to assess pain provocation from pressure applied to the lumbar spine segments [8]. Springing test has shown fair to good reliability (kappa 0.21–0.73) for assessment of segmental pain provocation [36]. Flexibility testing of muscles connecting to the pelvis and femur was also included where any difference in length or stiffness between the left and right sides was noted.

Manual muscle testing of muscles around the hip and knee joint, i.e., isometric tests where the examiner provides resistance to movement, was also included with the purpose of identifying differences between sides [8]. Muscle coordination, or recruitment pattern, was also assessed for prone hip extension, where the powerlifter lay prone and extended their hip with a straight knee, thus activating the hip extensors (m. gluteii and hamstrings) and the examiner assessed which muscle group contracted first [37].

Finally, a test of lumbopelvic movement control, “Waiter’s bow,” was included to examine whether the powerlifter could maintain a neutral position in their lumbar spine while flexing at the hips to approximately 50°. This test has shown acceptable inter- and intra-rater reliability [38].

#### Clinical diagnosis

After the subjective and physical examinations, the protocol was concluded with a section where the PT was prompted to summarize their findings in a final assessment based on the signs and symptoms of associated neurophysiological pain mechanisms and the specific impairments in body functions associated with the pain problem of the powerlifters. The first part of the assessment included whether the examiner considered the low back or hip pain problem to be associated with any movement restriction in a specific area/joint and if so, what direction was restricted and what structure, i.e., joint, muscle or nerve, was likely causing the restriction. The second part included an assessment of whether the pain problem was associated with an increase in movement (“give”)/lack of movement control in the area of symptoms and if so, what direction of movement and which body region. The third part included an assessment of the powerlifters’ tissue quality, i.e., whether the pain problem was associated with an ongoing tissue repair (i.e., inflammatory process) or not and whether the impairments in body function were adaptive (i.e., had a protective purpose to the pain problem), or maladaptive (i.e., were thought to be part of a movement behavior contributing to sustaining the pain problem). The fourth part included an assessment of the dominating neurophysiological pain mechanism, based on signs and symptoms from both the subjective and physical examination, where the examiner was asked to classify the pain as dominatingly nociceptive pain, peripheral neuropathic pain, or central sensitization syndrome (i.e., nociplastic pain) [39]. Assessment of neurophysiological pain mechanisms based on signs and symptoms from subjective and physical examinations has shown acceptable and excellent content validity, diagnostic accuracy (for nociceptive pain sensitivity 90.9%, specificity 91%, for peripheral neuropathic pain 86.3% and 96%, and for central sensitization 91.8% and 97.7%) and inter-rater reliability (Kappa 0.77–0.96) [9]. Previous studies on the reliability of different movement-based subclassification systems have shown moderate to good inter-reliability (Kappa = 0.40–0.80) [11].

Finally, the protocol prompted the examiner to formulate a clinical diagnosis consisting of the dominating neurophysiological pain mechanism and the primary findings relating to the specific impairments in body function that were associated with the powerlifter’s pain problem according to the assessments made during the examination.

### Phase two—evaluation of feasibility

In total, eight powerlifters were included. The powerlifter’s background characteristics are presented in Table 2.

#### Fulfilment of feasibility criteria

On average, each assessment took 55 (± 8) min. All the powerlifters (*n* = 8) were diagnosed according to the protocol without any of the PTs having to add items to the protocol, and when the PTs assessed the same powerlifters, they concluded the same clinical diagnosis of dominating neurophysiological pain mechanism and primary findings relating to the specific impairments in body function for all the powerlifters (*n* = 4). In 85% (11/13) of the powerlifters in whom pain was associated with performing the squat and/or deadlift, symptoms could be eased by altering the movement strategy. The most frequent alteration, which could ease pain during the exercises, involved different strategies/instructions that facilitated maintenance of a neutral position of the lumbar spine, i.e., moving away from provocative ranges of motion in the lumbopelvic area.

#### Agreement between PTs

After the assessment of the four powerlifters examined by both PTs, there was a substantial agreement regarding the clinical diagnosis of the dominating pain mechanism and associated specific impairments in body function of the powerlifters’ low back/hip pain. The diagnoses formulated by each PT for each of the four powerlifters are presented in Table 3.

**Table 3:** Clinical diagnosis of dominating pain mechanism and associated specific impairments in body function of the low back/hip pain of the four powerlifters (PL #1–4) assessed independently by two physical therapists. The assessments are presented divided by each physical therapist (PT #1 and 2)

	Physical therapist #1	Physical therapist #1
Powerlifter #1	Nociceptive mechanical pain. Restricted movement in flexion and internal rotation in right hip with possible articular origin	Nociceptive mechanical pain. Restricted movement in flexion and internal rotation in right hip with articular origin
Powerlifter #2	Nociceptive mechanical pain. Possible signs of peripheral neuropathic pain Increased give in lumbar extension. Activation of mm. obliquus eases pain during back squat	Nociceptive mechanical pain. Possible signs of peripheral neuropathic pain Increased give in lumbar extension
Powerlifter #3	Nociceptive mechanical pain Increased give in lumbar flexion, possibly associated with restricted movement in flexion in right hip. Forces thoracic spine in extension during back squat, thus increasing lumbar spine flexion	Nociceptive mechanical pain Forces thoracic spine in extension during back squat and lumbar spine flexion and pelvic posterior tilt when bracing the core
Powerlifter #4	Nociceptive inflammatory pain. Ongoing tissue reparation Overload on lower lumbar spine in flexion due to hyperextension in thoracolumbar spine, evident in back squat	Nociceptive mechanical pain. Ongoing tissue reparation Overload of lumbar spine, locally, due to hyperextension in thoracic spine evident in back squat

Both PTs made similar assessments for most items in the physical examination for each powerlifter. The items that diverged the most between PTs were the items related to observing and describing the powerlifters’ resting posture and manual muscle testing.

#### Suggested revisions for the protocol

In the “feasibility questionnaire” both PTs reported suggestions for improvements in the protocol. Based on the subjective examination of the powerlifters, the PTs suggested that more specific questions about the 24-h behavior of pain and the relationship between pain response and biomechanical factors should be added. Additionally, a question regarding previous examinations and treatments for the present pain disorder could be included.

Regarding the physical examination, the PTs suggested that several components could be added: a trial treatment, i.e., the option of performing and evaluating the results of a preliminary treatment to confirm/reject the clinical diagnosis. A section with specific palpation for pain provocation of joint structures and muscles. Additional assessments of adjacent joints (ankle, knee, and thoracic spine) and movement control tests of adjacent joints. Analysis of the bench press exercise in addition to the squat and deadlift. Quantification of muscle strength and flexibility, rather than just assessing side differences. In addition to the powerlifting exercises, the addition of a physical marker that the powerlifters choose themselves.

In the “feasibility questionnaire” both PTs further emphasized that the clinical diagnosis most often relied on the analysis of the provocative activities, i.e., the back squat and deadlift exercises, and the alteration of movement strategies in order to evaluate any decrease in symptoms were important.

There were some items that the PTs thought could be removed from the protocol because they were redundant. The specific assessments of the activities chosen in the PSFS were performed since all the powerlifters already had reported the powerlifting exercises as the activities that were most limited. Also, the separation of assessments of range of motion where the PT first assessed range of motion, and thereafter repeated the movements and assessed whether pain was provoked. Finally, to perform the neurological assessment on all powerlifters, despite not showing any signs of peripheral neuropathic pain in the subjective examination.

## Discussion

This is the first study to develop and evaluate the feasibility of a comprehensive clinical assessment protocol designed to explore the characteristics of powerlifters’ musculoskeletal pain problems in the low back and hip. Since very little was published regarding best practice to assess and examine powerlifters, the protocol was mainly based on literature and curricula on the assessment of musculoskeletal pain problems within physical therapy and specific literature related to injuries in powerlifting. The protocol was found to be feasible for use in forming a clinical diagnosis of powerlifters with low back and/or hip pain since the examination could be completed in approximately 1 h, and the two PTs consistently used it as the basis for their clinical diagnosis: i.e., they did not have to add tests or assessments to reach the clinical diagnosis. The PTs also made very similar assessments and clinical diagnoses, despite certain components (observation of posture and muscle testing) clearly differing between the assessors. Some aspects of the protocol should be further improved before conducting larger studies investigating the specific characteristics of pain problems/injuries in powerlifters.

In powerlifting, pain problems and/or injuries in the low back and/or hip region are prevalent, the problems mainly have a gradual onset and develop into chronic/recurrent problems and thus far, there is no convincing evidence that the pain problems are strongly associated to structural pathology [1, 14, 40]. With this in mind, the protocol was developed to include items that would allow for a clinical diagnosis describing powerlifters’ pain problem with emphasis on the associated dominating neurophysiological pain mechanism and the specific individual impairments in body function associated with the pain problem. Thereby providing direct information which could guide treatment. A clinical diagnosis centered on describing the individual’s pain problem from a movement and physical functioning standpoint, as opposed to seeking a purely pathoanatomical diagnosis, has been argued for by several clinicians and researchers [10, 12, 19, 22]. The main reason is that a pathoanatomical diagnosis does not provide the therapist with adequate information on what specific impairments in body function are associated with the specific individual seeking care; thus, it does not provide essential information to guide treatment [10, 12, 19, 22].

Since there is no previously documented clinical assessment protocol regarding the character of pain problems/injuries in powerlifters, the initial protocol consisted of a wide range of examination methods. As the goal of the examination was to provide a clinical diagnosis that could guide treatment it was necessary to include both a subjective examination and a physical examination. Due to the lack of consensus on what should be included in a physical examination, the items included in the protocol were based on the components most often included in the physiotherapeutic examination, i.e. observation/inspection, functional movements, active and passive joint mobility, muscle function, and neurological examination. However, some components were thought to be essential when assessing powerlifters, namely the specific examination of the powerlifting exercises, both regarding observation of movement strategies and if pain could be provoked or eased during performance and/or alteration of the powerlifters’ movement strategy. This component of the physical examination is recommended by Ryder [8] and Van Dillen et al. [32], who suggest that therapists start with an analysis of the most likely provocative movements/activities, and from these, further components in the examination are added. This specific component of the physical examination was also considered to be the most important for reaching the clinical diagnosis by the two PTs in the present study. Related to this component, the PTs also recommended that further movement control and muscle recruitment tests should be added to the protocol, in order to better understand which factors, contribute to the powerlifters’ individual movement strategy in the powerlifting exercises. However, it is notable that healthy powerlifters exhibit lower shoulder range of motion compared with individuals not participating in powerlifting [41], and that there seem to be no differences in performance of non-sport specific movement control tests between powerlifters with/without low back pain [42], thus potentially confounding the understanding of which factors affect individual movement strategies in the squat and/or deadlift exercises and the relevance of individual specific tests for the powerlifter’s pain problem.

Other aspects that were also suggested to be included in a revised protocol included more questions in the subjective examination regarding the behavior of pain in relation to activity, load, and rest and in the physical examination, the use of trial treatments, and more in-depth examination of adjacent joints. Trial treatments help either reject or confirm hypotheses and further examination of adjacent joints was suggested because the PTs believed that limited mobility in one joint could lead to compensation in a nearby/other joints/regions. This reasoning is also supported by Kaltenborn [21] and Van Dillen et al. [32]. The PTs also thought that assessments or tests that grade general weakness or tightness in muscles, not just the comparisons between the right and left sides, were lacking in the protocol. Adding these elements requires extensive use of medical equipment, such as dynamometers, and adding additional elements may increase the time for each examination, which should be carefully considered in relation to the benefits.

Several components were also not included in the protocol, although they are prevalent in the clinical setting, continuing/postgraduate curriculum, and course literature related to physical therapy. Two such components were palpation and specific motion-testing of the segmental range of motion and quality of end-feel in the spine. The reason for excluding these techniques from the protocol was that these techniques have been shown to be unreliable, with an intra- and inter-rater reliability of Kappa < 0.4 [43]. However, tests of the passive range of motion of the lumbar spine were included with the purpose of identifying pain provocative movements and assessing any substantial limitations in the range of motion of the spine as a whole. It can be argued that both the validity and reliability of these passive movement tests for establishing the dominating neurophysiological pain mechanism improve when they are used as pain provocation tests [3].

## Methodological considerations

A strength of the present study lies in the clinical relevance of including both PTs and powerlifters, i.e., the group of individuals whom the research will impact in the development and evaluation of a new protocol i.e., “participatory research” [44].

There are some limitations that should be noted. First, powerlifters with any signs of serious pathology were excluded from the study. Therefore, a revision to the protocol needs to systematically include questions regarding signs and symptoms of serious pathology. Second, only four powerlifters were examined by two PTs and an additional four powerlifters were examined by one of the PTs. The purpose of the recruitment was to include enough powerlifters to enable the evaluation of feasibility, thereby facilitating further revisions to the protocol. Therefore, no reliability analysis was intended or possible at this stage. Also, there is a risk that the included sample size of eight powerlifters could have been insufficient to provide enough variation of the powerlifter’s low back and/or hip pain problems in order to fully evaluate the feasibility of the protocol on an entirety of different pain problems that might exist. However, the sample size was deemed sufficient to evaluate the protocol for the feasibility criteria in the present study as a first step in the development and evaluation of the protocol.

Third, the initial protocol was drafted after a review of both scientific literature and PT curricula in the areas of examination and assessment of musculoskeletal disorders and sports injuries. However, it should be noted that while it is a limitation that the initial literature review did not conform to e.g., the Preferred Reporting Items for Systematic reviews and Meta-Analyses (PRISMA) guidelines, the review strategy was tailored for the purpose of identifying key research and practices to draft the protocol. Also, since the assessment and classification of injuries in powerlifting is a sparsely studied area there was not much specific information available. Therefore, a pragmatic approach to developing an assessment protocol was thought to be based on the intended purpose and population for which it is to be used.

Fourth, the protocol included some orthopedic special tests, i.e., tests with the goal of ruling in or out specific pathology and/or reproducing symptoms in a specific localization. The tests assessed neurology (sensitivity to touch, isometric muscle tests, reflex tests, and mechanosensitivity testing), pain provocation of the lumbar spine (springing test), and hip joint morphology (Craig’s test). Neurology testing was included to identify signs of peripheral neuropathic pain and although these tests have shown a wide variation in diagnostic accuracy for identifying radiculopathy[30] they remain a component in guidelines in the assessment of low back pain [45]. Pain provocation tests such as the springing test add information related to both the pain mechanism and localization of the pain, in this case by applying an overpressure to a segment of the spine [36]. Although it has so far only been evaluated for reliability it was thought to have clinical value for the reasons mentioned. Craig’s test was included to evaluate the femoral version angle and is a reliable and valid test for this purpose [34, 35]. Since the powerlifting squat and deadlift are movements that heavily engage the hip joints and musculature, Craig’s test was thought to possibly be an important part of an assessment in order to evaluate the powerlifter’s lifting stance in relation to the results of Craig’s test. It should be noted that Craig’s test has not been evaluated for this purpose and none of the PTs highlighted Craig’s test as a vital component when determining their diagnosis.

Fifth, it can also be argued that the protocol might need some degree of training to be useful for PTs other than those included in the present study. In this study, the PTs had similar educational backgrounds, had the same postgraduate education, and were familiar with both the powerlifting exercises and the assessment and treatment of powerlifters with different musculoskeletal disorders. Since both PTs emphasized that the examination of the squat and deadlift exercises was essential for their clinical diagnosis, it can be assumed that this might be a prerequisite for therapists to use the protocol.

Sixth, the protocol had simple and clear instructions, although the positions and specific test techniques for the items in the physical examination were not specified in detail. The main reasoning for this was to rely on the knowledge of the PTs to assess the specific physical functions included but also to steer away from creating a specific concept of subclassification. Concepts such as “movement system impairments” [19] or “mechanical diagnostic therapy” [46], which typically require PTs to invest time and money to buy in the whole concept of theoretical frameworks as well as specific examination techniques. A strength of this approach was that it was thought to increase the clinical relevance of the protocol and lower the prerequisite for the protocol to be implemented in further research and clinical practice. However, as with all instruments, trial runs are recommended before implementation, and the PTs in this study also stated that using the protocol to document findings as they performed the examination was tedious and distracted from their clinical reasoning process.

Seventh, the examination of sport-specific movements needs to be addressed. Both PTs suggested that the bench press should have been included in the protocol, given that the back squat and deadlift were specifically examined. The bench press often involves hyperextension of the lower back, placing stress on that region, similar to the other two lifts. While the protocol did include a section on “Physical Markers” where activities associated with pain and/or limitations were assessed, such as the squat and deadlift, with adjustments made to the lifting technique if pain was present, a mandatory inclusion of the bench press could provide additional insights. This could help guide exercise modifications and enable continued sports participation. Additionally, during the examination of the squat and deadlift, all powerlifters used a 40-kg load. This was chosen for practical reasons, primarily to limit the time required to complete the protocol. However, it is important to note that lifting mechanics can change with increasing loads [41] and therefore also possibly symptoms. Consequently, a range of weights, tailored to each individual based on the loads they report as pain-provoking, should be considered when assessing powerlifters during any of the powerlifting exercises.

## Conclusions

This study showed that it was feasible to use the novel comprehensive clinical assessment protocol to formulate a clinical diagnosis of low back and/or hip pain in powerlifters including neurophysiological pain mechanisms, and impairments in body functions. The inclusion of the squat and deadlift exercises in the physical examination also seems especially important when making a clinical diagnosis of powerlifters with low back/hip pain. However, before larger studies are performed, the assessment protocol should be evaluated in a larger number of subjects to determine whether there is further need for revision and to investigate the reliability of the clinical diagnosis achieved after using the protocol.

## Supplementary Information

Additional file 1. Comprehensive clinical assessment protocol.

## Data Availability

The datasets used and/or analyzed during the current study are available from the corresponding author upon reasonable request.

## Abbreviations

PT: Physical therapist
SLR: Straight leg raise
PSFS: Patient-specific functional scale
