# Supplementary material for: Development of a comprehensive clinical assessment protocol for low back and hip pain in powerlifters: a feasibility study
Source: Pilot Feasibility Stud. 2024 Dec 12;10:150. doi: 10.1186/s40814-024-01579-0 (PMC11636030; doi:10.1186/s40814-024-01579-0)
Supplement: Supplementary file 1 — Additional file 1. Comprehensive clinical assessment protocol. [file 40814_2024_1579_MOESM1_ESM.docx]

## Patient Background Questionnaire (filled out by the patient)

| **Code number________** | Have you signed the informed consent form?  YES / NO | Age_______  Height_______  Weight_________  Sex_________ |
| --- | --- | --- |
| Do you currently experience pain or discomfort in the lumbar/pelvic/hip area that hinders your powerlifting training/competition?  YES / NO  If YES, what is your main complaint? (e.g., pain in the groin)………………………………………………  When do you experience your pain or discomfort? (e.g., when performing back squats)  .……………………………………………………...…………………………… | | |
| Have you had the pain/discomfort for more than 3 months?  YES / NO | | |
| Did your symptoms start gradually or suddenly (acute)?  GRADUALLY / SUDDENLY (ACUTE)  How would you describe the onset of your symptoms? (e.g., during squat training, leisure time, slipping last winter, etc.) ………………………………………………………………………………………………………………………………………………………………………………………………………...................................................... | | |
| How many years of experience do you have of powerlifting training (squat, bench press, and deadlift)? (number of years)………………………….. | | |
| How many times per week do you train? (number of sessions)  Squats......  Bench Press......  Deadlift...... | | |
| How many hours per week do you spend on powerlifting training?.................... | | |
| What are your personal records (kg)?  Squat with belt..................................Squat without belt.......................  Deadlift with belt...............................Deadlift without belt.....................  Bench Press.................. | | |

## Clinical assessment protocol for individuals with hip/low back pain

| Code number………… | Do they have pain/discomfort? YES / NO |
| --- | --- |
| **SUBJECTIVE EXAMINATION** | **Filled out by PT during examination** |
| Where does it hurt/do you have discomfort (point)? | HIP / LOW BACK |
| How long have the complaints been going on? | Number of months………………… |
| Does the pain vary during the day?  When is the pain at its worst? | MORNING / AFTERNOON / EVENING / NIGHT / ONLY DURING MOVEMENT |
| Pain intensity and characteristics for each body part | VAS 7 days per body part……………  VAS right now per body part…………  ACHING / STABBING / CRAMPING / STINGING / RADIATING / CUTTING / BURNING / SMARTING / NUMBING |
| Pain behavior (which movements/activities provoke/ease your pain) | Provoking =  Easing = |
| Patient Specific Functional Scale (PSFS) Identify primary functional impairment:  "Due to your hip/lower back problems, what activities can you no longer perform to the extent you could before the problems started?" 0-10  0 = cannot perform the activity at all  10 = can perform the activity to the extent before the problems started (no activity limitation) | Activity 1 =  Activity 2 =  Activity 3 = |
| Pain Avoidance Behavior:  Does the individual stop doing the movement/activity that causes pain? | YES / NO |
| What are the patients own thoughts and beliefs about the pain? |  |

| **PHYSICAL EXAMINATION** | **Filled out by PT during examination** |
| --- | --- |
| **OBSERVATION in standing** | **Anterior and posterior view:** |
| Paraspinal muscles (encircle which side is "smaller") | LEFT / RIGHT / SYMMETRICAL |
| SIPS (encircle the most cranial side) | LEFT / RIGHT / SYMMETRICAL |
| SIAS (encircle the most cranial side) | LEFT / RIGHT / SYMMETRICAL |
| Crista iliaca height, feet toghether (encircle the most cranial side) | LEFT / RIGHT / SYMMETRICAL |
| Femur rotation, left | MEDIAL / NEUTRAL / LATERAL |
| Femur rotation, right | MEDIAL / NEUTRAL / LATERAL |
| Knee, left | VARUS / NEUTRAL / VALGUS |
| Knee, right | VARUS / NEUTRAL / VALGUS |
| Ankle, left | PRONATED / NEUTRAL / SUPINATED |
| Ankle, right | PRONATED / NEUTRAL / SUPINATED |
|  | **Lateral view:** |
| Lumbar lordosis | INCREASED / NETRAL / DECREASED |
| Thoracic kyphosis | INCREASED / NETRAL / DECREASED |
| Pelvic tilt, left | ANTERIOR / NEUTRAL / POSTERIOR |
| Pelvic tilt, right | ANTERIOR / NEUTRAL / POSTERIOR |
| Hip joint, left | FLEXION / EXTENSION /NEUTRAL |
| Hip joint, right | FLEXION / EXTENSION /NEUTRAL |
| **PERIPHERAL NERVE TESTS** |  |
| Neurological integrity, patient in supine.  Difference right/left (note/encircle which side is impaired/changed) |  |
| Achilles reflex | LEFT / RIGHT / SYMMETRICAL |
| Quadriceps reflex | LEFT / RIGHT / SYMMETRICAL |
| Sensory perception (light touch)(encircle which dermatome is imparired/changed) | LEFT - L1 / L2 / L3 / L4 /L5 / S1  RIGHT - L1 / L2 / L3 / L4 /L5 / S1  SYMMETRICAL |
| Muscle strength (isometric) | LEFT - L1 / L2 / L3 / L4 /L5 / S1  RIGHT - L1 / L2 / L3 / L4 /L5 / S1  SYMMETRICAL |
| Mechanosensitivity. Patient in supine. |  |
| Straight leg raise, left | POSITIVE / NEGATIVE |
| Straight leg raise, right | POSITIVE / NEGATIVE |
| **PHYSICAL MARKERS** |  |
| Examination of the difficulties/activity limitations that the powerlifter stated in the PSFS (subjective examination)   1. Does the movement provoke pain? 2. Can pain be alleviated with alteration of movement pattern? 3. If yes, what alteration of the movement was performed? | Activity 1:…………………………  1.………………………………….  2.………………………………….  3.……………………………….… |
|  | Activity 2:…………………………  1.………………………………….  2.………………………………….  3.……………………………….… |
|  | Activity 3:…………………………  1.………………………………….  2.………………………………….  3.……………………………….… |
| Observation of starting position of the back squat with barbell (circle what undesired movement or position is occuring) | THORACOLUMBAR EXTENSION / THORACIC FLEXION / THORACIC EXTENSION / LUMBAR FLEXION / LUMBAR EXTENSION / PELVIS POSTERIOR TILT / PELVIC ANTERIOR TILT / HIP FLEXION / HIP EXTENSION / HIP LATERAL ROTATION / HIP MEDIAL ROTATION |
| Examination of the barbell back squat   1. Does the movement provoke pain? 2. Can pain be alleviated with alteration of movement pattern? 3. If yes, what alteration of the movement was performed? | 1.………………………………….  2.………………………………….  3.……………………………….… |
| Observation of starting position of the barbell deadlift (circle what undesired movement or position is occuring) | THORACOLUMBAR EXTENSION / THORACIC FLEXION / THORACIC EXTENSION / LUMBAR FLEXION / LUMBAR EXTENSION / PELVIS POSTERIOR TILT / PELVIC ANTERIOR TILT / HIP FLEXION / HIP EXTENSION / HIP LATERAL ROTATION / HIP MEDIAL ROTATION |
| Examination of the barbell deadlift   1. Does the movement provoke pain? 2. Can pain be alleviated with alteration of movement pattern? 3. If yes, what alteration of the movement was performed? | 1.………………………………….  2.………………………………….  3.……………………………….… |
| **PAIN PROVOCATION** |  |
| Which movement direction provokes recognizable pain? (circle the movement direction that provokes pain) |  |
| Low back  Active movements: Tested in standing as the starting position. Instruct patient to perform the movements. | FLEXION / EXTENSION / LATERAL FLEXION LEFT / LATERAL FLEXION RIGHT / ROTATION LEFT / ROTATION RIGHT |
| Low back  Passive movements: Tested in side lying as a starting position. | FLEXION / EXTENSION / LATERAL FLEXION LEFT / LATERAL FLEXION RIGHT / ROTATION LEFT / ROTATION RIGHT |
| Sprining test/PA test L1-L5:  (encircle the painful level). Tested in prone with lumbar spine in a neutral position as a starting position. | L1 / L2 / L3 / L4 / L5 |
| Hip  Active movements: Tested in standing as the starting position with hip in zero position. Instruct patient to perform the movements with one leg at a time. | LEFT LEG:  FLEXION / EXTENSION / LATERAL ROTATION / MEDIAL ROTATION / ADDUCTION / ABDUCTION  RIGHT LEG:  FLEXION / EXTENSION / LATERAL ROTATION / MEDIAL ROTATION / ADDUCTION / ABDUCTION |
| Hip  Passive movements: Tested in supine and prone, hip in zero position as a starting position. | LEFT LEG:  FLEXION / EXTENSION / LATERAL ROTATION / MEDIAL ROTATION / ADDUCTION / ABDUCTION  RIGHT LEG:  FLEXION / EXTENSION / LATERAL ROTATION / MEDIAL ROTATION / ADDUCTION / ABDUCTION |
| **Summary of pain provocation tests:** |  |
| Where does the movement that provokes pain occur? | LOW BACK / HIP LEFT / HIP RIGHT |
| Which movement direction? (indicate one direction) | …………………………….. |
| Which examination (active/passive) is your assessment based on? | …………………………….. |

| **ACTIVE AND PASSIVE MOVEMENTS (RANGE OF MOTION)** |  |
| --- | --- |
| Angular range of motion: Differences between right/left side (note/encircle which side has limited range of motion) |  |
| Low back  Active movements: Tested in standing as the starting position. Instruct patient to perform the movements. | FLEXION / EXTENSION / LATERAL FLEXION LEFT / LATERAL FLEXION RIGHT / ROTATION LEFT / ROTATION RIGHT |
| Low back  Passive movements: Tested in side lying as a starting position. | FLEXION / EXTENSION / LATERAL FLEXION LEFT / LATERAL FLEXION RIGHT / ROTATION LEFT / ROTATION RIGHT |
| Hip  Active movements: Tested in standing as the starting position with hip in zero position. Instruct patient to perform the movements with one leg at a time. | LEFT LEG:  FLEXION / EXTENSION / LATERAL ROTATION / MEDIAL ROTATION / ADDUCTION / ABDUCTION  RIGHT LEG:  FLEXION / EXTENSION / LATERAL ROTATION / MEDIAL ROTATION / ADDUCTION / ABDUCTION |
| Hip  Passive movements: Tested in supine and prone, hip in zero position as a starting position. | LEFT LEG:  FLEXION / EXTENSION / LATERAL ROTATION / MEDIAL ROTATION / ADDUCTION / ABDUCTION  RIGHT LEG:  FLEXION / EXTENSION / LATERAL ROTATION / MEDIAL ROTATION / ADDUCTION / ABDUCTION |
| Anteversion/retroversion hip:  Craig’s test., patient in prone position.  The hip ante-/retroversion is estimated when the trochanter major is judged to be furthest out in the room (most laterally) seen from the patient (10° inward rotation = normal/neutral) | LEFT LEG  ANTEVERSION / RETROVERSION / NEUTRAL  RIGHT LEG  ANTEVERSION / RETROVERSION / NEUTRAL |
| **MUSCLE FUNCTION** |  |
| **Flexibility**  Note difference between right-left sides  Circle which side is tight and if possible also assess whether; K=structurally short muscle or S=increased stiffness muscle. Any testing method/body position is allowed, but crucial to perform bilaterally! |  |
| Iliopsoas | RIGHT / LEFT - K / S |
| Gluteus | RIGHT / LEFT - K / S |
| Hamstrings | RIGHT / LEFT - K / S |
| Rectus femoris | RIGHT / LEFT - K / S |
| Tensor fascia latae | RIGHT / LEFT - K / S |
| Adductors | RIGHT / LEFT - K / S |
| Piriformis | RIGHT / LEFT - K / S |
| **Muscle strength**  Note difference between right-left sides  Circle which side is weaker and YES on the side where pain response occurs, if applicable. Any testing method/body position, but crucial to perform bilaterally! |  |
| Iliopsoas (tensor fascia late, sartorius, rectus femoris) | RIGHT - YES / LEFT - YES |
| Gluteus maximus: | RIGHT - YES / LEFT - YES |
| Gluteus medius/minimus | RIGHT - YES / LEFT - YES |
| Hamstrings | RIGHT - YES / LEFT - YES |
| Quadriceps | RIGHT - YES / LEFT - YES |
| Tensor fascia latae | RIGHT - YES / LEFT - YES |
| Adductors | RIGHT - YES / LEFT - YES |
| Hip external rotators | RIGHT - YES / LEFT - YES |
| Hip internal rotators | RIGHT - YES / LEFT - YES |
| Obliques | RIGHT - YES / LEFT - YES |
| **Muscle recruitment** **pattern** – prone hip extension.  Tested in prone position with extended knee.  Circle the muscle that activates first/initiates the movement. | LEFT:  GLUTEUS / HAMSTRINGS  RIGHT:  GLUTEUS / HAMSTRINGS |
| **Movement control test** – Waiter’s bow  Tested in standing position.  Eccentric phase, forward flexion of the hip to 50° with neutral lumbar spine?  Concentric phase, hip extension to neutral position with neutral lumbar spine? | ECCENTRIC PHASE:  YES / NO  CONCENTRIC PHASE:  YES / NO |

| **FINAL ASSESSMENT** | **Filled out by PT after examination** |
| --- | --- |
| Is there a movement restriction associated with the pain | YES / NO |
| If YES, in which area? | HIP / LOW BACK |
| Which/what movement direction(s)? | ……………………………………… |
| Which structure is causing the restriction? | ARTICULAR / MUSCULAR / NERVE / UNCLEAR |
| Do you assess that the pain is associated with increased mobility (give)? | YES / NO |
| If YES, in which area? | HIP / LOW BACK |
| Which/what movement direction(s)? | ……………………………………… |
| Physiotherapist's assessment of current tissue and healing mechanism | ONGOING HEALING / NO HEALING |
| Are the issues/condition adaptive or maladaptive (here and now)? | MALADAPTIVE / ADAPTIVE |
| Physiotherapist's assessment of the current dominant pain mechanism | NOCICEPTIVE MECHANICAL / NOCICEPTIVE INFLAMMATORY / NOCICEPTIVE ISCHEMIC / PERIPHERAL NEUROPATHIC / NOCIPLASTIC |
| **DIAGNOSIS** |  |
| Formulate a description the patients pain/complaint through a Functional Diagnosis, i.e., a connection between findings in history and examination. | Free text answer.  Be sure to indicate if nociceptive issues are associated with reduced strength, decreased flexibility, increased stiffness (area), unfavorable muscle recruitment patterns. If peripheral neuropathic issues are associated with impaired sensation, muscle strength, loss of reflex, and/or increased mechanosensitivity, and for nociplastic pain, if which symptoms of increased central sensitization are present. |
|  | |

| On what basis (signs and symptoms in the subjective and physical examination) did you formulate your diagnosis? Refer, in free text, to the specific items in the protocol. |
| --- |
|  |
